# Supplementary material for: TFEB regulates lysosomal positioning by modulating TMEM55B expression and JIP4 recruitment to lysosomes
Source: Nat Commun. 2017 Nov 17;8:1580. doi: 10.1038/s41467-017-01871-z (PMC5691037; doi:10.1038/s41467-017-01871-z)
Supplement: Supplementary file 1 — Supplementary Information [file 41467_2017_1871_MOESM1_ESM.pdf]

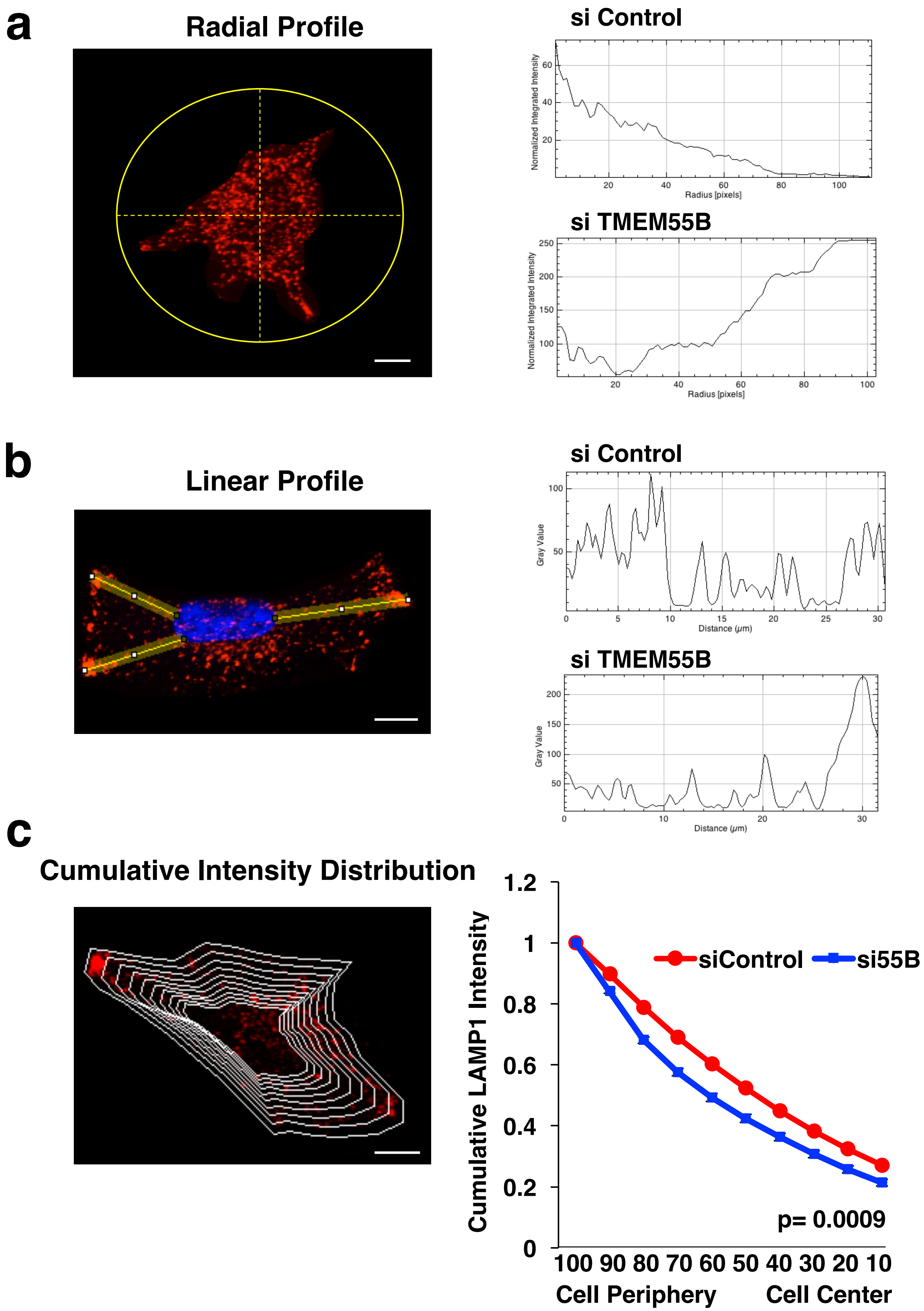
a
Radial Profile
si Control
Normalized Integrated Intensity
Radius [pixels]
si TMEM55B
Normalized Integrated Intensity
Radius [pixels]
b
Linear Profile
si Control
Gray Value
Distance (μm)
si TMEM55B
Gray Value
Distance (μm)
c
Cumulative Intensity Distribution
Cumulative LAMP1 Intensity
siControl
si55B
p= 0.0009
100 90 80 70 60 50 40 30 20 10
Cell Periphery
Cell Center

Supplementary Figure 1: Lysosome positioning quantification. (**a**) Radial profile depiction and quantification of LAMP-1 signal in individual siControl and siTMEM55B HeLa cells. (**b**) Linear profile depiction and quantification of LAMP-1 signal in individual siControl and siTMEM55B HeLa cells. Linear profile width of 10 pixels=3 *μ*m. (**c**) Cumulative intensity distribution of LAMP-1 signal in siControl and siTMEM55B HeLa cells. siControl n=37, siTMEM55B n=37. Error bars denote s.e.m. Note that despite the quantification method, the bulk of LAMP-1 signal in siControl cells is perinuclear whereas the bulk of LAMP-1 signal in siTMEM55B cells is distributed towards the cell periphery. Scale bars, 10 μm.

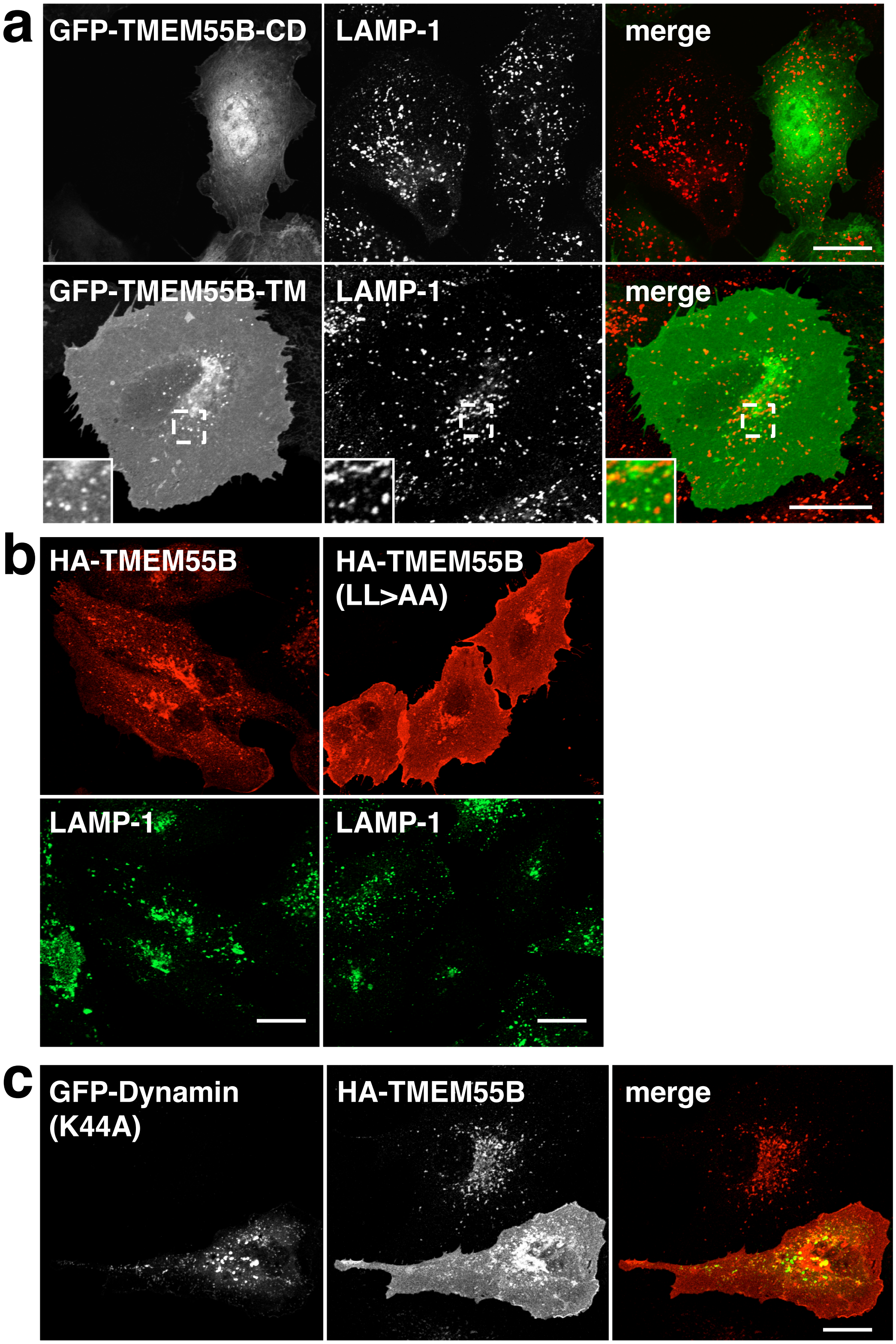

Supplementary Figure 2: TMEM55B CD is required for trafficking of TMEM55 to lysosomes. **(a)** ARPE-19 cells transfected with either GFP-TMEM55B CD or GFP-TMEM55B TM. Cells were fixed, permeabilized, and immunostained with antibodies against LAMP-1. Insets represent a 2.5-fold magnification of the indicated area. **(b)** ARPE-19 cells transfected with either 3xHA-TMEM55B WT or mutant (LL>AA). Cells were fixed, permeabilized, and immunostained with antibodies against HA and LAMP-1. **(c)** ARPE-19 cells co-transfected with 3xHA-TMEM55B WT and mutant GFP-Dynamin (K44A). Cells were fixed, permeabilized, and immunostained with antibodies against HA (red). Scale bars, 20 μm.

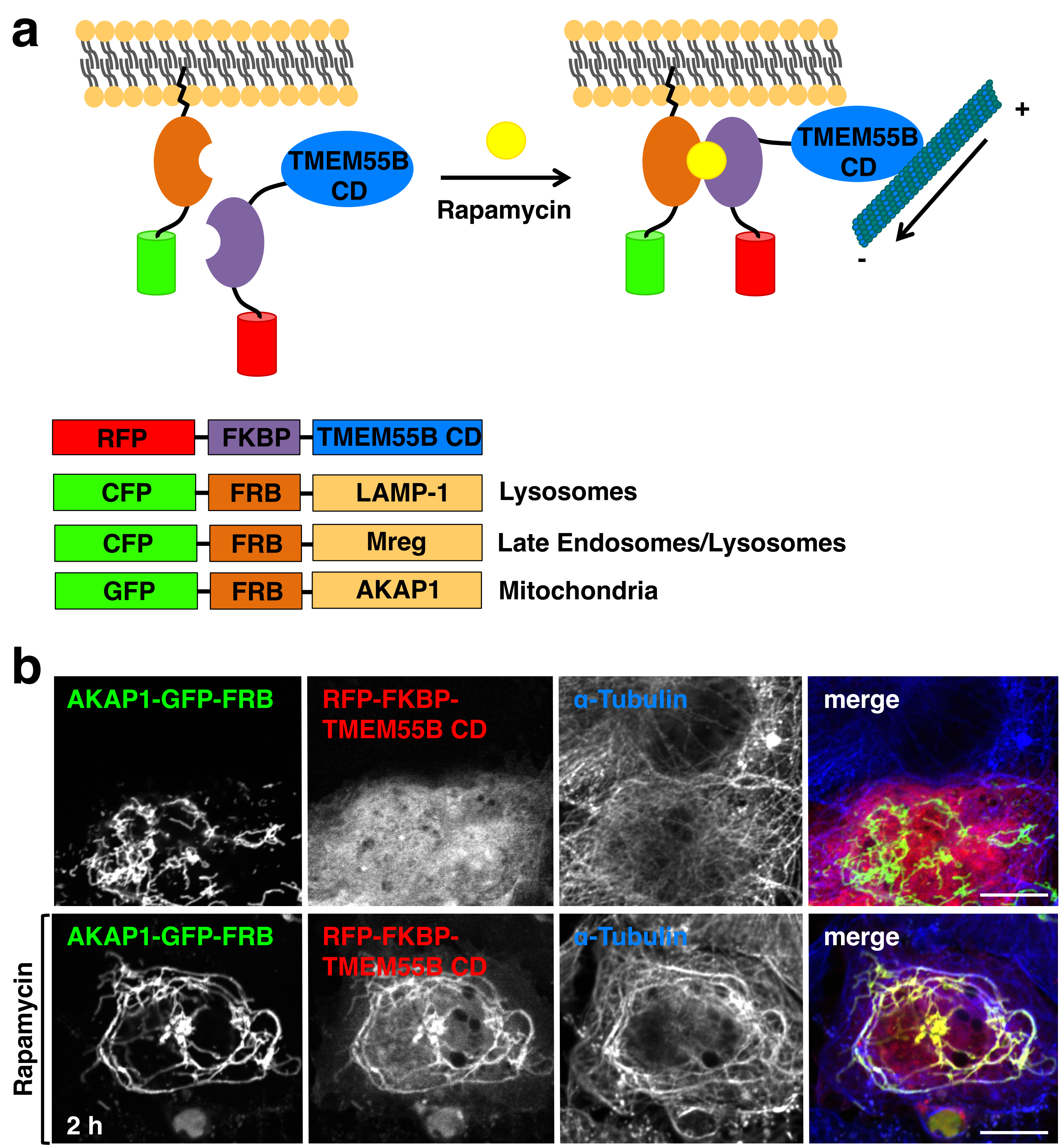

Supplementary Figure 3: Inducible heterodimerization assay to determine TMEM55B activity. (**a**) Schematic model of Rapamycin-induced heterodimerization of cytosolic RFP-FKBP-TMEM55B CD and FRB-C/GFP tagged membrane anchors; LAMP-1 lysosomal membrane anchor, melanoregulin (Mreg) endosomal membrane anchor, AKAP1 outer mitochondria membrane anchor. Recruitment of TMEM55B CD initiates microtubule re-organization and retrograde transport of tagged membranes towards the cell center. (**b**) ARPE-19 cells co-expressing AKAP1-GFP-FRB and RFP-FKBP-TMEM55B CD for 24 h. Cells were incubated in 200 nM Rapamycin for the indicated time. Cells were fixed, permeabilized, and stained with antibodies against α-tubulin. Scale bar, 10 µm.

a

		RFP Pull-down					
		Control		RFP-FKBP-TMEM55B CD -Rapamycin		RFP-FKBP-TMEM55B CD +Rapamycin	
Accession	Gene ID	Unique Peptides	Coverage	Unique Peptides	Coverage	Unique Peptides	Coverage
O60271	SPAG9	0	0%	116	33%	161	34%
P11279	LAMP1	0	0%	9	6%	43	24%

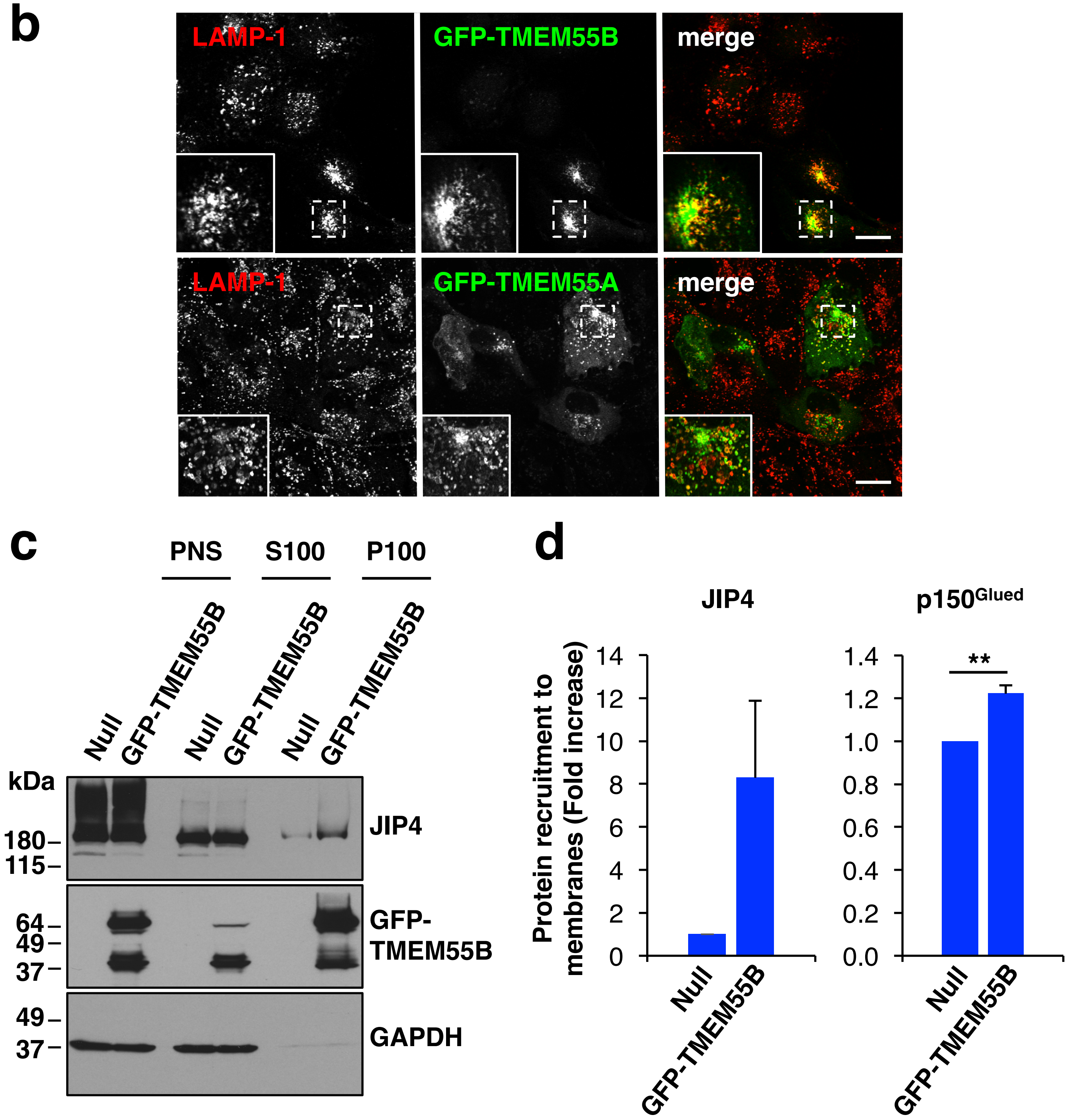

Supplementary Figure 4: TMEM55A expression does not affect lysosome positioning.
(a) Mass spectrometry analysis of RFP pull-downs from ARPE-19 cells co-expressing RFP-FKBP-TMEM55B and LAMP-1-CFP-FRB with and without 200 nM Rapamycin treatment. TMEM55B CD pull-down bound to JIP4 (SPAG9) compared to non-transfected cells regardless of membrane association. As a control for membrane recruitment, LAMP-1 specifically interacts with TMEM55B CD after Rapamycin treatment. (**b**) ARPE-19 cells expressing GFP-TMEM55B (top) or GFP-TMEM55A (bottom) for 24 h. Cells were fixed, permeabilized, and immunostained with antibodies against LAMP-1. Scale bar, 20 µm. (**c**) Immunoblot of subcellular fractions from HeLa cells infected with adenovirus expressing GFP-TMEM55B or Adenovirus Null. Cell lysates were centrifuged to obtain postnuclear supernatant (PNS), and high-speed centrifuged supernatant (S100) and membranes (P100). (**d**) Quantification of JIP4 and $p150^{Glued}$ recruitment to P100 fractions expressed as a fold increase compared to cell infected with adenovirus Null from three independent experiments. Error bars denote s.e.m. p-value calculated using two-tailed t-test **P<0.01

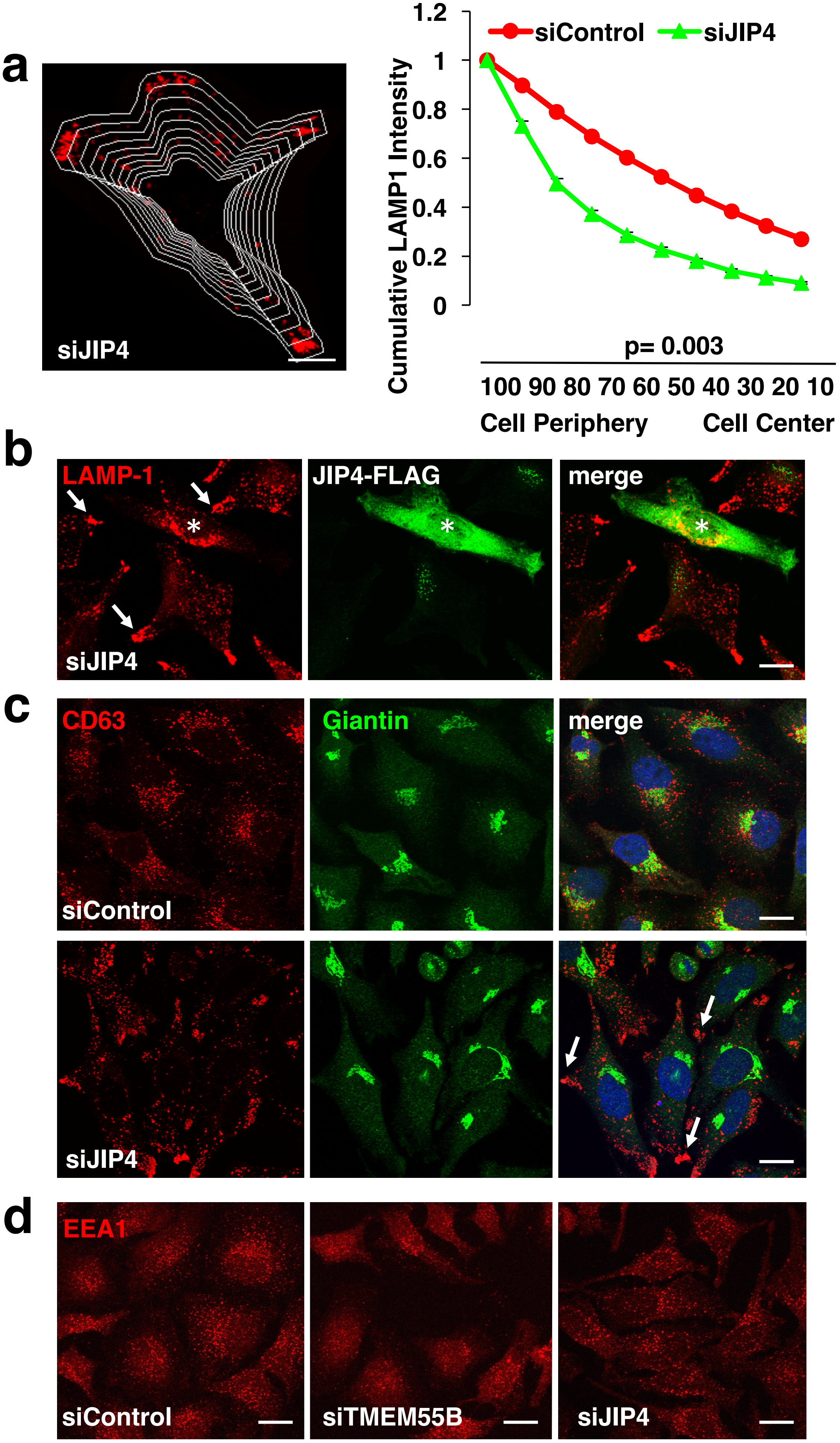

a
siJIP4
1.2
1
0.8
0.6
0.4
0.2
0
Cumulative LAMP1 Intensity
siControl
siJIP4
p= 0.003
100 90 80 70 60 50 40 30 20 10
Cell Periphery
Cell Center
b
LAMP-1
JIP4-FLAG
merge
siJIP4
c
CD63
Giantin
merge
siControl
siJIP4
d
EEA1
siControl
siTMEM55B
siJIP4

Supplementary Figure 5: TMEM55B and JIP4 depletion does not affect positioning of other membrane compartments. (**a**) Cumulative intensity distribution of LAMP-1 signal in siControl and siTMEM55B HeLa cells. siControl n=37, siJIP4 n=30 error bars denote s.e.m. **(b)** HeLa cells treated with JIP4 siRNA were transfected with JIP4-Flag for 24 h. Cells were fixed, permeabilized, and immunostained with antibodies against LAMP-1 and JIP4. Asterisks indicate JIP4-Flag expressing cells. (**c-d**) HeLa were cells treated with TMEM55B, JIP4, or Control siRNAs. Cells were fixed, permeabilized, and immunostained with antibodies against CD63 and Giantin (**c**) or EEA1 (**d**). Scale bars, (**a**) 10μm, (**b-d**) 20 μm.

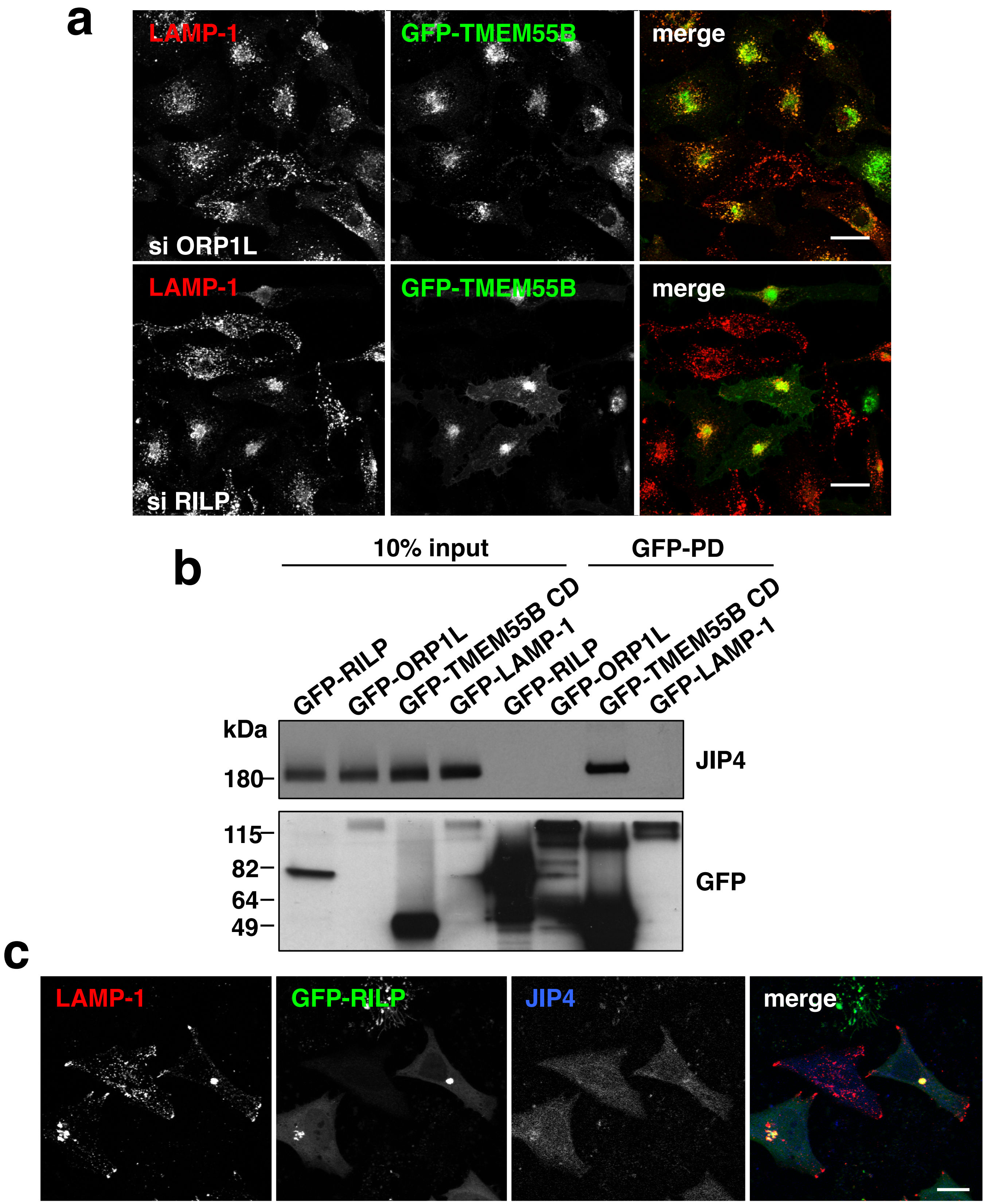

Supplementary Figure 6: TMEM55B drives lysosomal clustering independently of RILP. **(a)** HeLa cells treated with ORP1L or RILP siRNA and infected with Ad-GFP-TMEM55B for 24 h. Cells were fixed and immunostained with antibodies against LAMP-1. (**b**) Immunoblot of GFP pull-down from ARPE-19 cell lysates transfected with GFP-RILP, GFP-ORP1L, GFP-TMEM55B CD, or GFP-LAMP-1 + HA-TMEM55B FL. (**c**) HeLa cells expressing GFP-RILP for 24 h. Cells were fixed and immunostained with antibodies against LAMP-1 and JIP4. Scale bars, 20 µm.

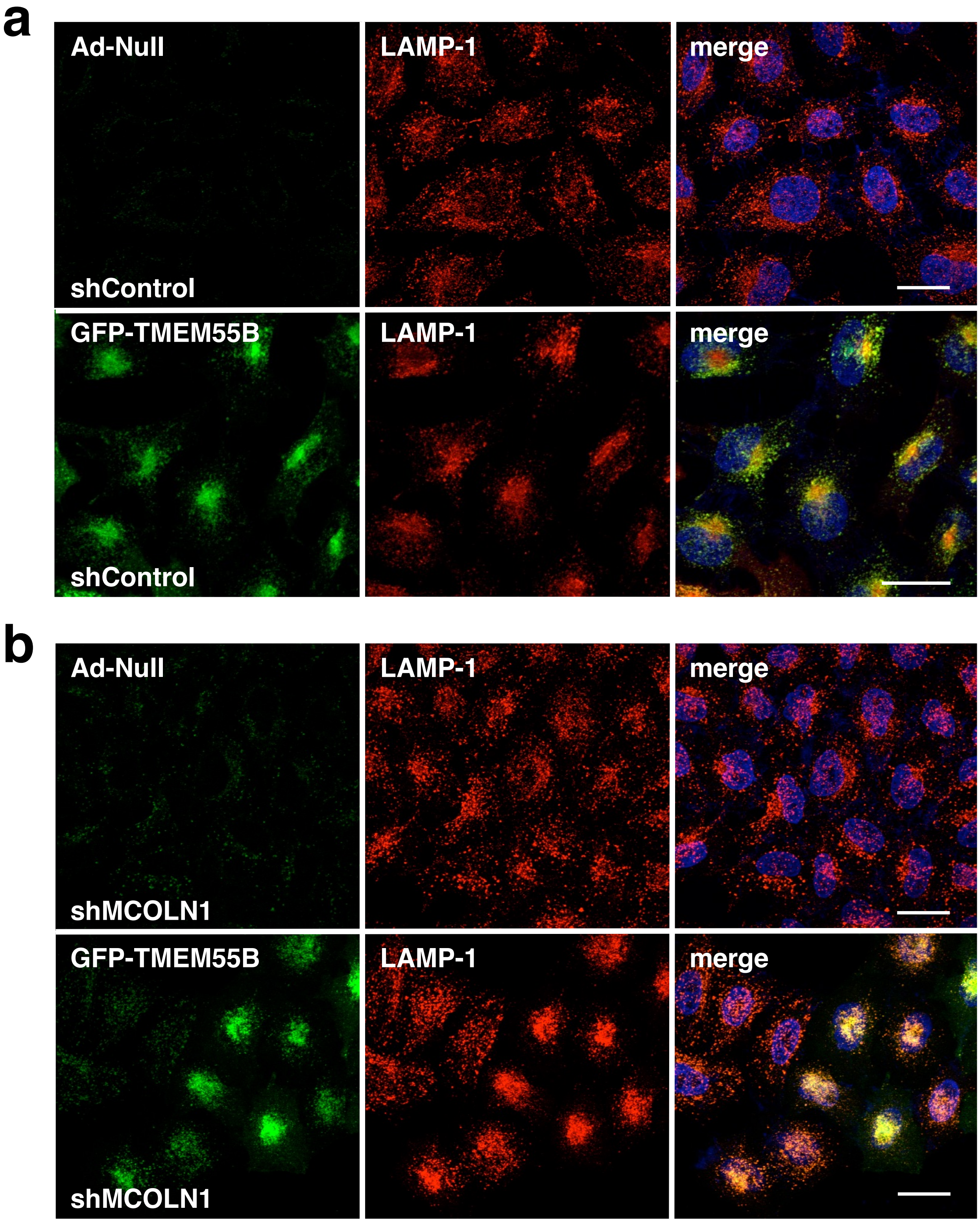

Supplementary Figure 7: MCOLN1 is not required for TMEM55B-induced lysosomal clustering. HeLa shControl (**a**) or shMCOLN1 (**b**) cells were infected with adenovirus expressing GFP-TMEM55B for 24 h. Cells were fixed, permeabilized, and immunostained with antibodies against LAMP-1 (red) and DAPI (blue). Scale bars, 20 µm.

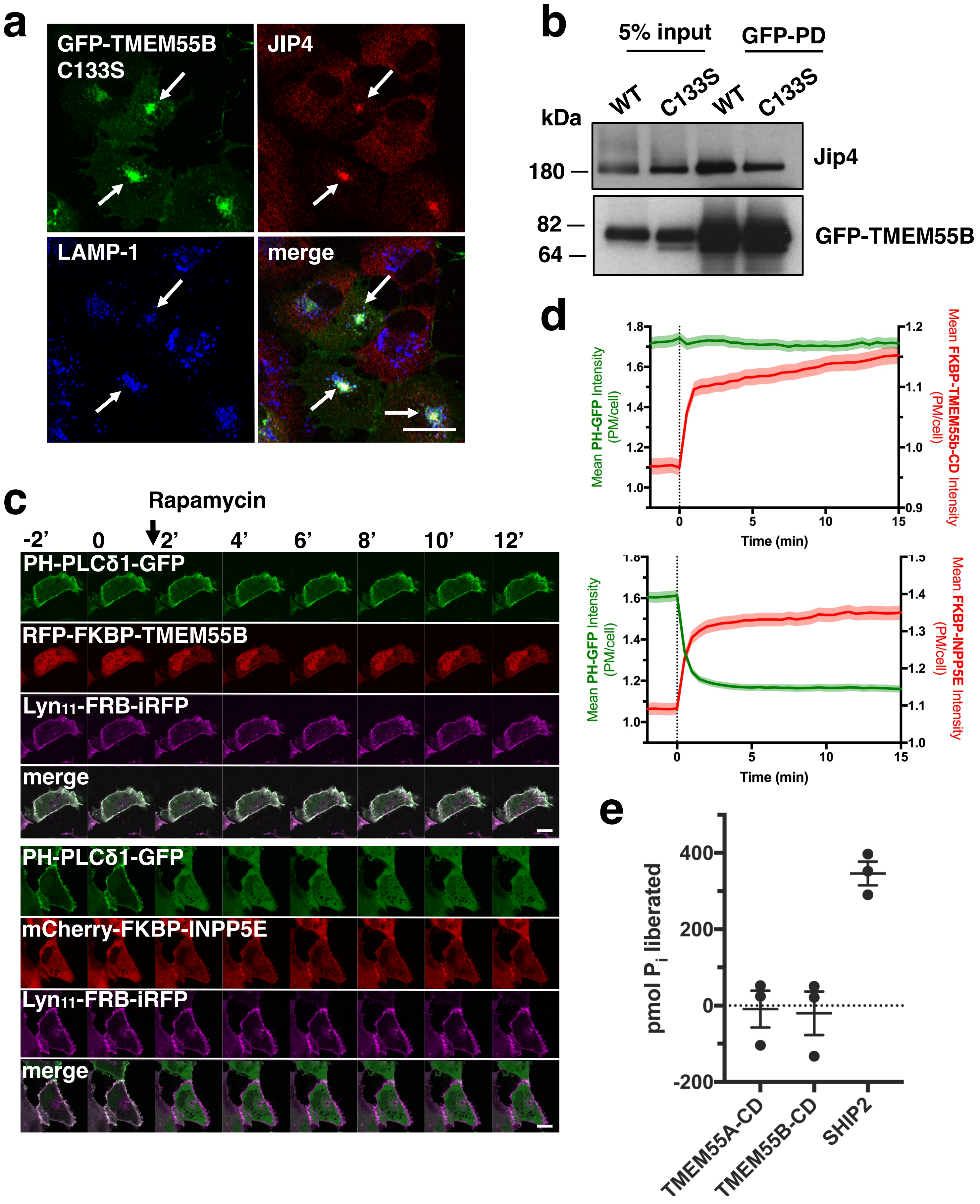
a
GFP-TMEM55B C133S
JIP4
LAMP-1
merge
b
5% input
GFP-PD
WT
C133S
WT
C133S
kDa
180
82
64
Jip4
GFP-TMEM55B
c
Rapamycin
-2'
0
2'
4'
6'
8'
10'
12'
PH-PLCδ1-GFP
RFP-FKBP-TMEM55B
Lyn11-FRB-iRFP
merge
PH-PLCδ1-GFP
mCherry-FKBP-INPP5E
Lyn11-FRB-iRFP
merge
d
Mean PH-GFP Intensity (PM/cell)
Mean FKBP-TMEM55b-CD Intensity (PM/cell)
Time (min)
Mean PH-GFP Intensity (PM/cell)
Mean FKBP-INPP5E Intensity (PM/cell)
Time (min)
e
pmol Pi liberated
TMEM55A-CD
TMEM55B-CD
SHIP2

Supplementary Figure 8: TMEM55B is not an active PIP_2 phosphatase. (**a)** GFP-TMEM55B carrying a point mutation (C133S) in its putative Cx5R catalytic motif was expressed in ARPE-19 cells for 24 h. (a) Cells were fixed, permeabilized, and immunostained with antibodies against LAMP-1 and JIP4. Note arrows indicating clustering of LAMP-1 and recruitment of JIP4 to clustered membranes. Scale bar, 20 µm. (**b**) Immunoblot of GFP pull-down from lysates of HeLa cells transfected with GFP-TMEM55B or GFP-TMEM55B-C133S. (**c**) mRFP-FKBP-TMEM55B (top panels, red) or mCherry-FKBP-INPP5E positive control (bottom panels, red) were acutely recruited to the plasma membrane via rapamycin-induced dimerization with Lyn_{11}-FRB-iRFP (both panels, magenta) in COS7 cells. No loss of plasma membrane PIP_2 detected with PH-PLCδ1-GFP (both panels, green) was observed with TMEM55B-CD recruitment, whereas INPP5E induced a complete loss of PM PIP_2. (**d**) quantification of FKBP (red) or PH-PLCδ1-GFP (green) at the plasma membrane (defined by a mask derived from the Lyn_{11}-FRB-iRFP signal) relative to whole cell fluorescence. Scale bar, 10 μm. Data are means ± s.e.m. of 63 cells from three independent experiments. (**e**) TMEM55A CD and TMEM55B CD lack phosphatase activity. 250 ng of each recombinant enzyme was assayed against 20 μM short-chain PtdIns-4,5-P_2 substrates for 15 min at 37˚C using the malachite green phosphate release assay. Data are grand means from three experiments performed in duplicate, and show means ± sem. Scale bar, 10 µm.

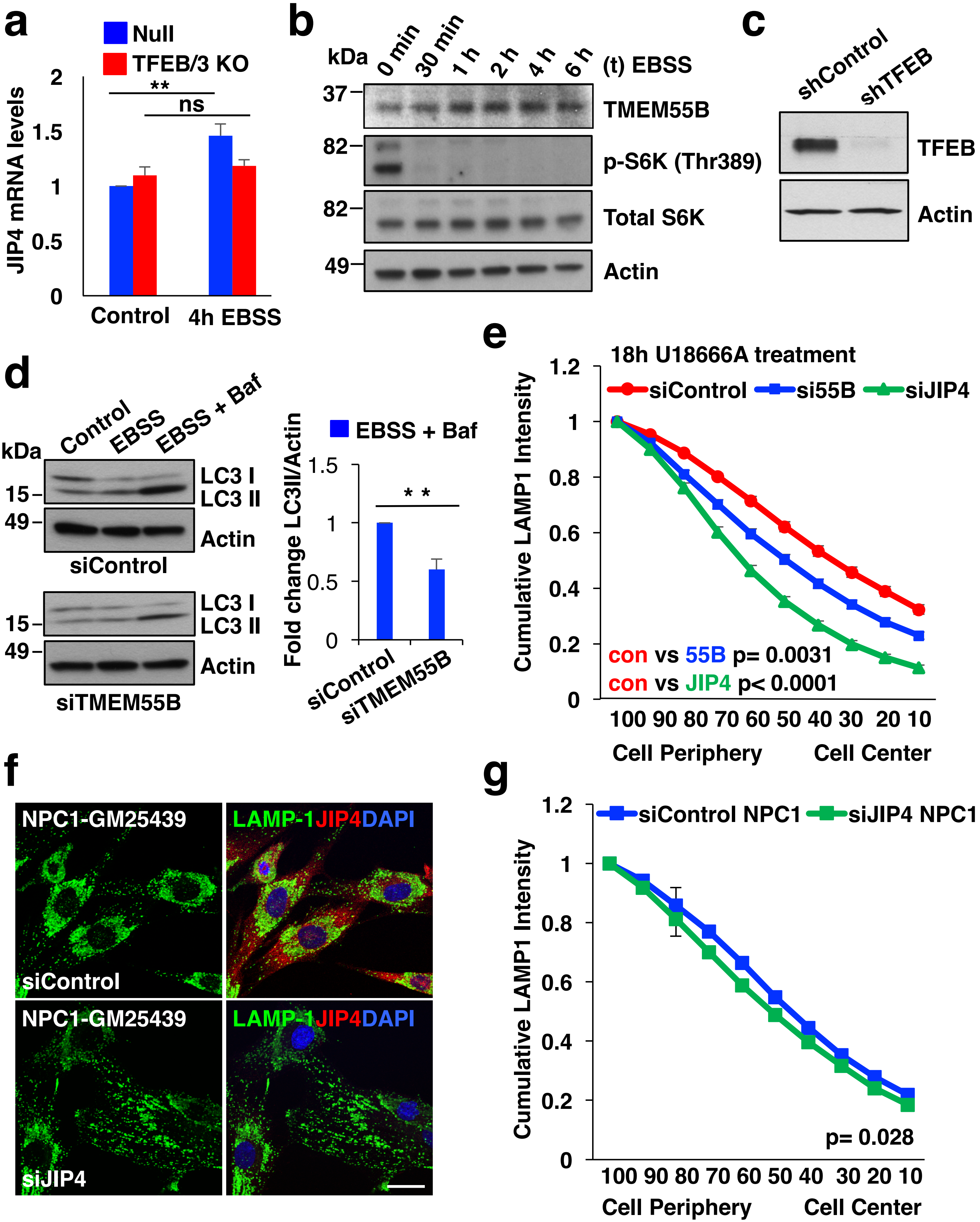

a
Null
TFEB/3 KO
JIP4 mRNA levels
**
ns
Control
4h EBSS
b
kDa
0 min
30 min
1 h
2 h
4 h
6 h
(t) EBSS
37
TMEM55B
82
p-S6K (Thr389)
82
Total S6K
49
Actin
c
shControl
shTFEB
TFEB
Actin
d
kDa
Control
EBSS
EBSS + Baf
15
LC3 I
LC3 II
49
Actin
siControl
15
LC3 I
LC3 II
49
Actin
siTMEM55B
Fold change LC3II/Actin
EBSS + Baf
* *
siControl
siTMEM55B
e
18h U18666A treatment
siControl
si55B
siJIP4
Cumulative LAMP1 Intensity
con vs 55B p= 0.0031
con vs JIP4 p< 0.0001
100 90 80 70 60 50 40 30 20 10
Cell Periphery
Cell Center
f
NPC1-GM25439
LAMP-1JIP4DAPI
siControl
NPC1-GM25439
LAMP-1JIP4DAPI
siJIP4
g
siControl NPC1
siJIP4 NPC1
Cumulative LAMP1 Intensity
p= 0.028
100 90 80 70 60 50 40 30 20 10
Cell Periphery
Cell Center

Supplementary Figure 9: TFEB controls lysosome positioning following starvation.
(a) Relative quantitative real-time PCR analysis of JIP4 mRNA transcript levels from null-or TFE3/TFEB knockout MEFs, untreated or starved in EBSS for 4 h (mean ± s.e.m. of the RNA fold change of indicated JIP4 normalized to GAPDH) n=5 ($^{**}P$=0.001) from three independent experiments. (**b**) Immunoblot of lysates from HeLa cells starved in EBSS for indicated times. (**c**) Immunoblot of lysates from HeLa cells treated with control shRNA or shRNA to TFEB. (**d**) Immunoblot and quantification of $LC3_{II}$/Actin ratios from siControl or siTMEM55B treated HeLa cells starved in EBSS 4 h with 100 nM bafilomycin for 2 h. Quantified results are fold increase of $LC3_{II}$/Actin from siControl after starvation and bafilomycin treatment and data are presented as mean ± s.e.m. using two-tailed *t*-test $^{**}P$=0.00068, n=3. (**e**) Cumulative intensity distribution of LAMP-1 signal in siControl, siTMEM55B, or siJIP4 HeLa cells incubated with 10 µM U18666A for 18 h. siControl n=33, siTMEM55B n=30, siJIP4 n=29. Error bars denote s.e.m. (**f**) NPC1 patient (GM25439) fibroblasts depleted of JIP4 with RNAi. Cells were fixed, permeabilized, and immunostained with antibodies against LAMP-1, JIP4, and DAPI. Scale bar, 20 µm. (**g**) Cumulative intensity distribution of LAMP-1 signal in siControl or siJIP4 NPC1 (GM03123) patient fibroblasts cells siControl n=21 siJIP4 n=20. Error bars denote s.e.m.

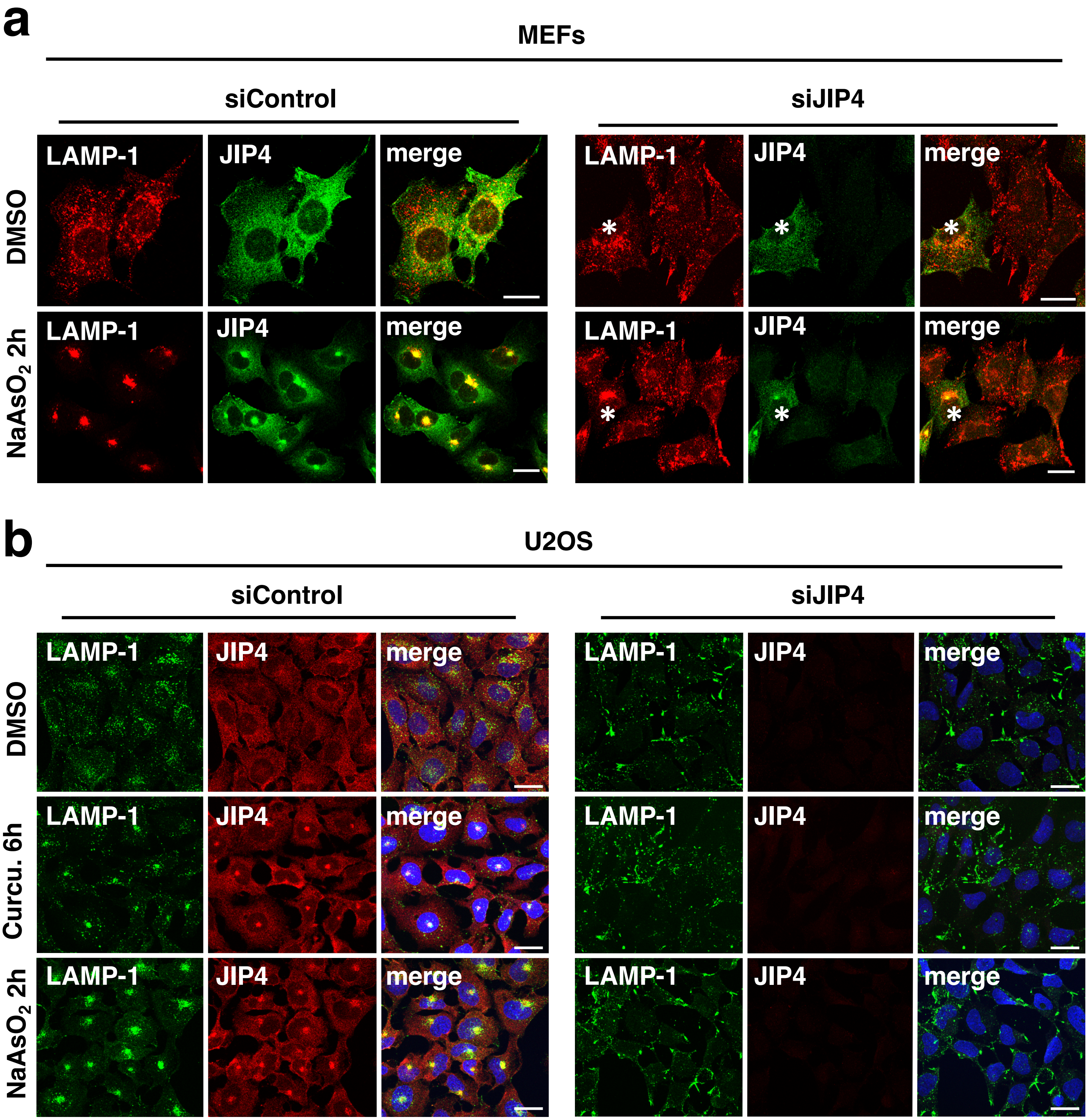

Supplementary Figure 10. Acute stress induced lysosomal clustering is dependent on TMEM55B/JIP4. (**a**) Control or JIP-4 depleted MEFs were treated with 150 uM $NaAsO_2$ for 2 h and immunostained with antibody against LAMP-1 and JIP4. Asterisks indicate cells in which JIP4 depletion was not complete. **(b)** Control or JIP-4 depleted U2OS cells were treated with curcumin or $NaAsO_2$ for the indicated times. Cells were fixed, permeabilized and immunostained with antibodies against LAMP-1, JIP4, and DAPI. Scale bars, 20 µm.

Figure 4a

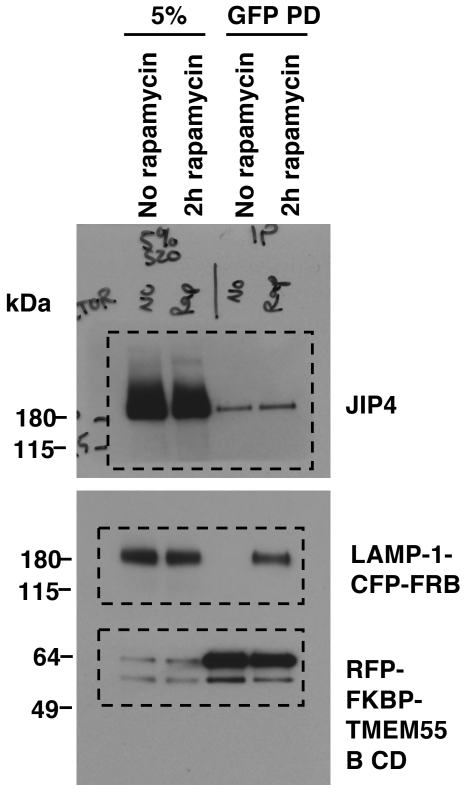

Figure 4b

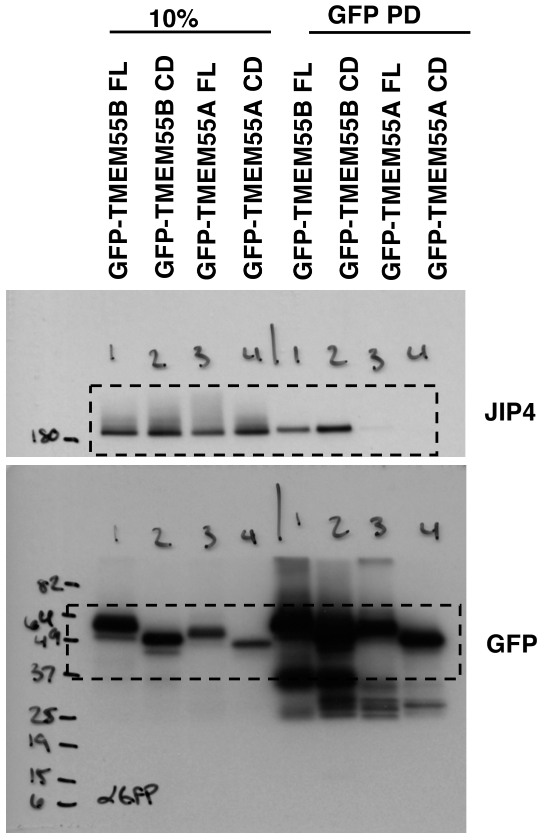

Figure 4c

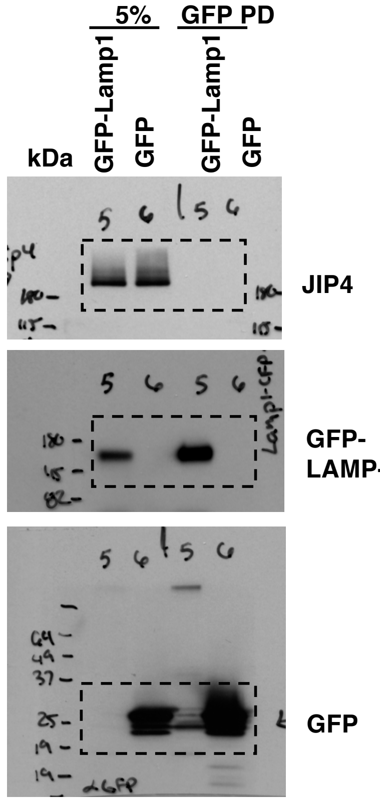

Figure 4d

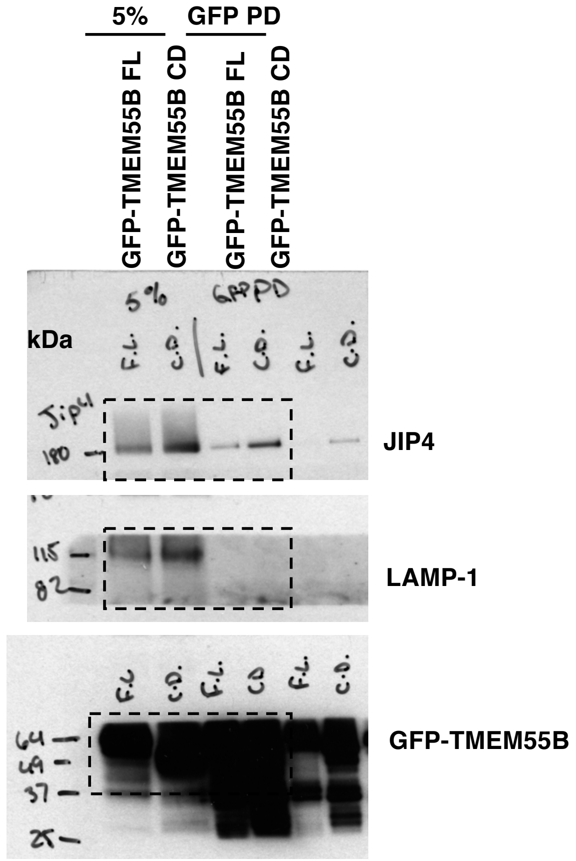

Figure 4e

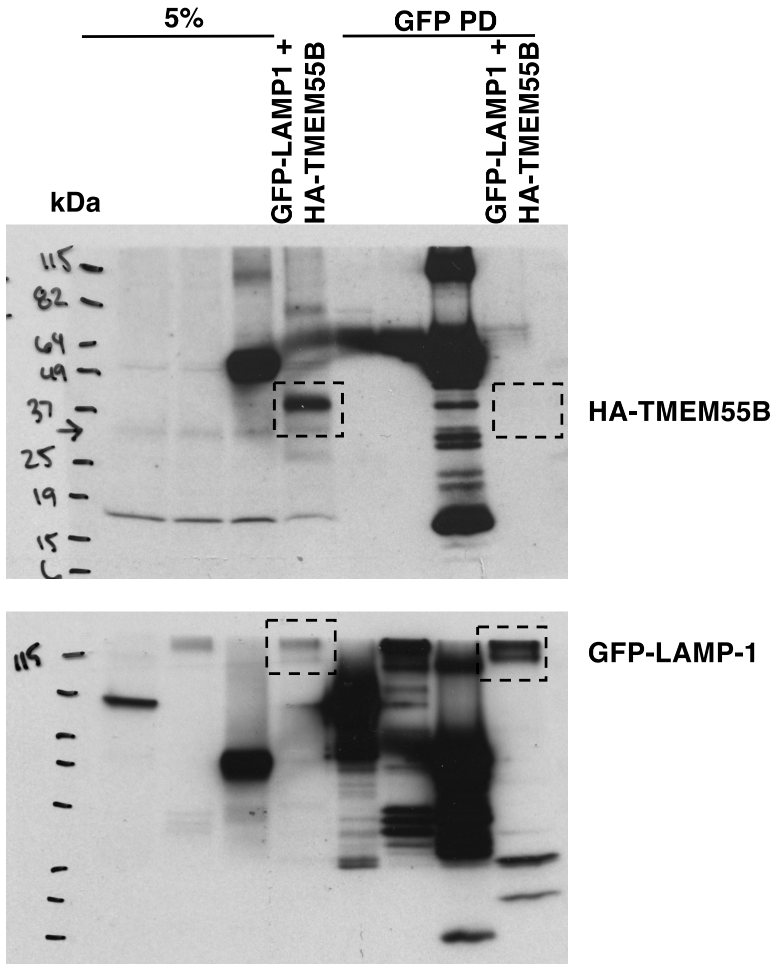

Figure 6b

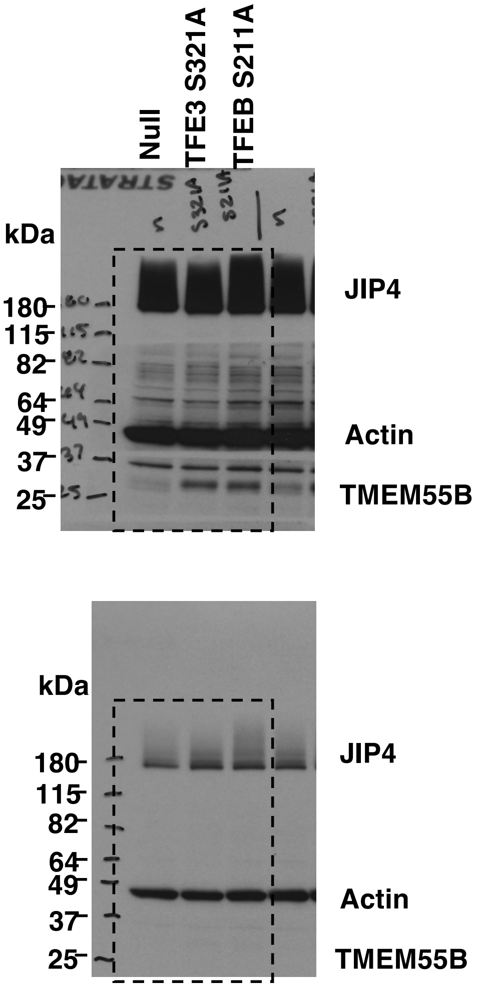

Figure 6k

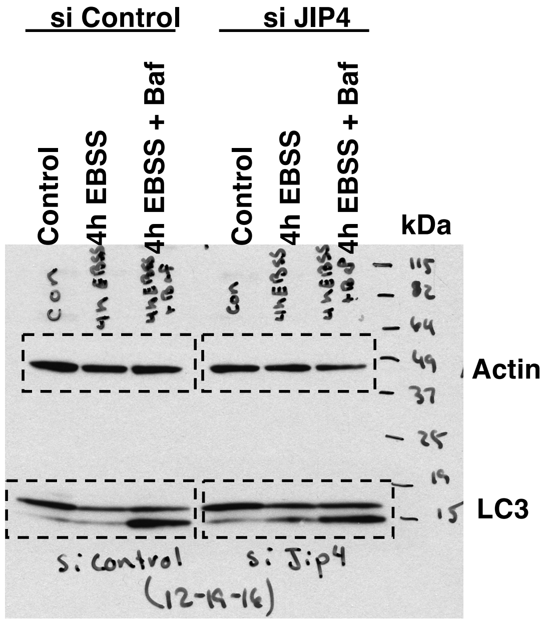

Figure 7b

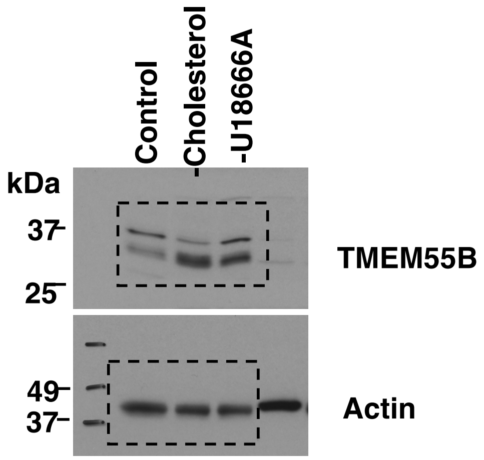

Figure 7g

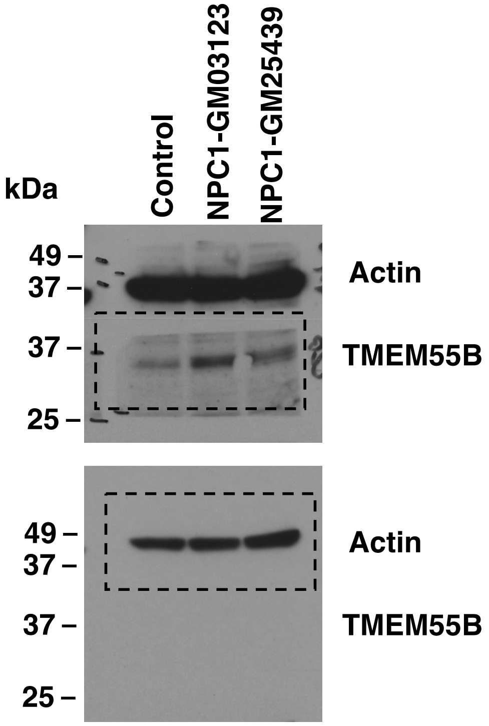

Figure 8f

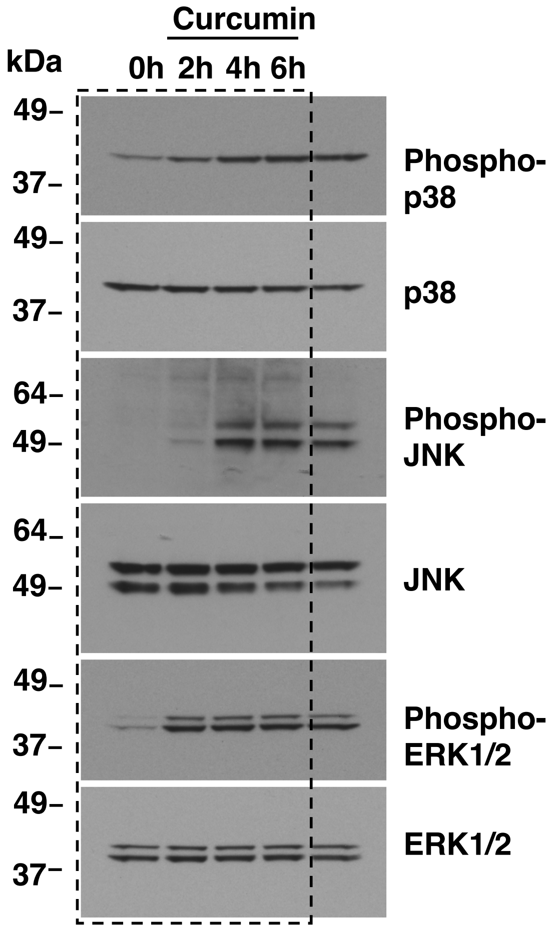

Figure 8e

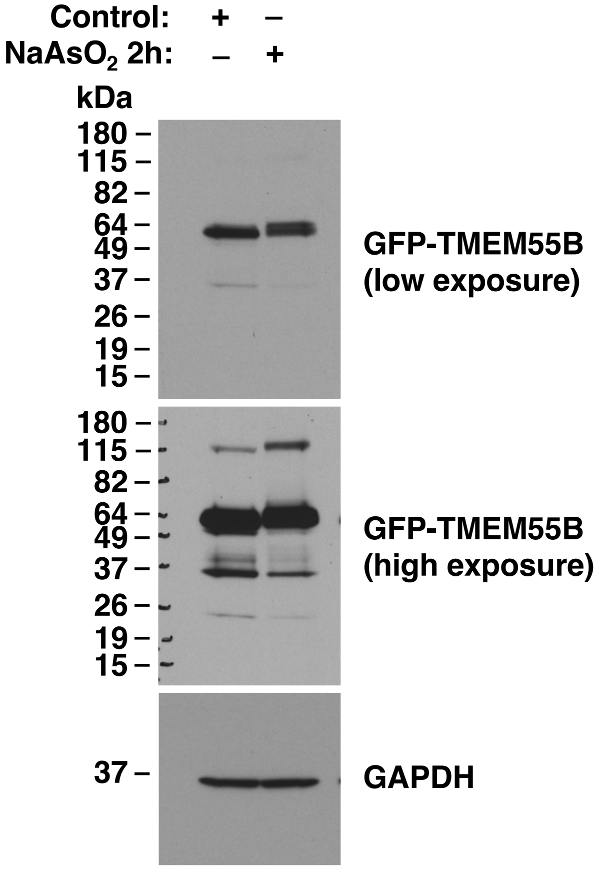

Supplementary Figure 11: Uncropped scans of immunoblots included in main figures.

Supplementary Table 1: List of antibodies used in this study

Target	Producer; Catalog Number	Western Blot Dilution	Immuno-fluorescence Dilution
**Lamp-1**	Developmental Studies Hybridoma Bank; H4A3	1:2000	1:3000
**HRS**	Novus Biological; NB100-55249		1:800
**EEA1**	BD Biosciences; 610456		1:500
**CD63**	BD Biosciences; 556019		1:2000
**TMEM55B**	Biorbyt; orb140078	1:500	
**Actin**	BD Biosciences; 612656	1:10000	
**a-Tubulin**	Sigma-Aldrich; T9026		1:500
**JIP4**	Cell Signaling Technology; 5519	1:2000	1:100
**TFE3**	Sigma-Aldrich; HPA023881		1:2000
**Phos S6K (Thr389)**	Cell Signaling Technology; 9205	1:1000	
**S6K**	Cell Signaling Technology; 2708	1:2000	
**GFP**	Roche; 11814460001	1:2000	
**HA clone 16B12**	Biolegend; 901501	1:2000	1:2000
**LC3**	Sigma-Aldrich; L7543	1:2000	1:300
**Giantin**	Covance; PRB114C		1:1000
**GAPDH**	Thermo-Fisher;AM4300	1:50000	
**p150-glued**	BD Biosciences; 610474	1:5000	1:250
**p38 MAPK**	Cell Signaling Technology; 9212	1:2000	
**Phospho-p38 MAPK (Thr180/Tyr182)**	Cell Signaling Technology; 9211	1:2000	
**SAPK/JNK**	Cell Signaling Technology; 9252	1:1000	
**Phospho-SAPK/JNK (Thr183/Tyr185)**	Cell Signaling Technology; 9255	1:500	
**p44/42 MAPK (Erk1/2)**	Cell Signaling Technology; 9102	1:2000	
**Phospho-p44/42 MAPK (Erk1/2) (Thr202/Tyr204)**	Cell Signaling Technology; 4377	1:2000	

Secondary antibodies

**Goat anti-rabbit IgG-Alexa Fluor 555**	Thermo-Fisher; A-21428		1:1000
**Goat anti-mouse IgG-Alexa Fluor 555**	Thermo-Fisher; A-21422		1:1000
**Goat anti-rabbit IgG-Alexa Fluor 647**	Thermo-Fisher; A-21244		1:1000
**Goat anti-rabbit IgG-Alexa Fluor 488**	Thermo-Fisher; A-11008		1:1000
**anti-mouse IgG-HRP**	Cell Signaling Technology; 7076S	1:5000	
**anti-rabbitt IgG-HRP**	Cell Signaling Technology; 7074S	1:5000	
