# Supplementary material for: TFEB regulates lysosomal positioning by modulating TMEM55B expression and JIP4 recruitment to lysosomes
Source: Nat Commun. 2017 Nov 17;8:1580. doi: 10.1038/s41467-017-01871-z (PMC5691037; doi:10.1038/s41467-017-01871-z)
Supplement: Supplementary file 3 — Description of Additional Supplementary Files [file 41467_2017_1871_MOESM3_ESM.pdf]

Description of Additional Supplementary Files

File Name: Supplementary Movie 1
Description: Recruitment of TMEM55B CD to lysosomes induces rapid clustering in the perinuclear region. Time-lapse imaging of ARPE-19 cells expressing mRFP-FKBP-TMEM55B-CD and Mreg-FRB-HA-CFP. Rapamycin was 3 added 3 min after the beginning of the recording. Note the rapid redirection of lysosome trafficking towards the cell center upon recruitment of TMEM55B CD to lysosome membranes after rapamycin. Images were acquired every 7 sec. Video speed 7 fps.
